# Brain Uptake of Folate Forms in the Presence of Folate Receptor Alpha Antibodies in Young Rats: Folate and Antibody Distribution

**DOI:** 10.3390/nu15051167

**Published:** 2023-02-25

**Authors:** Natasha Bobrowski-Khoury, Jeffrey M. Sequeira, Edward V. Quadros

**Affiliations:** 1 School of Graduate Studies, SUNY Downstate Health Sciences University, Brooklyn, NY 11203, USA; 2 Department of Medicine, SUNY Downstate Health Sciences University, Brooklyn, NY 11203, USA

**Keywords:** folate, folate receptor alpha, levofolinate, brain uptake, autism, cerebral folate deficiency

## Abstract

In a rat model, following exposure to rat folate receptor alpha antibodies (FRαAb) during gestation, FRαAb accumulates in the placenta and the fetus and blocks folate transport to the fetal brain and produces behavioral deficits in the offspring. These deficits could be prevented with folinic acid. Therefore, we sought to evaluate folate transport to the brain in young rat pups and determine what effect FRαAb has on this process, to better understand the folate receptor autoimmune disorder associated with cerebral folate deficiency (CFD) in autism spectrum disorders (ASD). When injected intraperitoneally (IP), FRαAb localizes to the choroid plexus and blood vessels including the capillaries throughout the brain parenchyma. Biotin-tagged folic acid shows distribution in the white matter tracts in the cerebrum and cerebellum. Since these antibodies can block folate transport to the brain, we orally administered various folate forms to identify the form that is better-absorbed and transported to the brain and is most effective in restoring cerebral folate status in the presence of FRαAb. The three forms of folate, namely folic acid, D,L-folinic acid and levofolinate, are converted to methylfolate while L-methylfolate is absorbed as such and all are efficiently distributed to the brain. However, significantly higher folate concentration is seen in the cerebrum and cerebellum with levofolinate in the presence or absence of FRαAb. Our results in the rat model support testing levofolinate to treat CFD in children with ASD.

## 1. Introduction

Folate in its many reduced forms fulfills the requirement for this vitamin in cellular metabolic processes. Folic acid (PGA), the oxidized chemical form, is used in most vitamin pills but is readily converted to the reduced methyltetrahydrofolate (MTHF) during intestinal absorption and translocation into circulation [1,2,3]. At higher doses, some PGA will be transported into circulation unconverted, and this cannot be utilized in folate-dependent pathways without first converting into tetrahydrofolate (THF). However, the efficiency of conversion will depend on the dihydrofolate reductase (DHFR) activity in specific tissues. DHFR activity varies widely in tissues and in the same organ of different species [4,5]. The jejunum, where most folate is absorbed, contains relatively high DHFR and other folate pathway enzymes, as is evident from the rapid conversion of PGA to MTHF. This form accounts for over 90% of circulating folate. In all organs, MTHF is the primary source for DHFR to generate THF. Higher amounts of unmetabolized PGA associated with daily intake and its potential deleterious effects have been reported in some population-based studies [6,7]. Behavioral deficits ranging from abnormal ultrasonic vocalizations to hyperactivity and memory deficits as well as structural alterations in sphingomyelin and phosphatidylcholine distribution in the neocortex and hippocampus have been associated with even a moderate increase in PGA intake in mouse models [8,9,10,11]. This may be particularly significant if one is treating daily with pharmacologic doses of the vitamin that could potentially result in high amounts of PGA in circulation. What remains unanswered, in this controversial observation and the claims of its deleterious effects, is the lack of evidence of PGA interfering with any of the folate pathway enzymes [12,13]. MTHF being the major form of folate both intra- and extra-cellularly, would be the primary contributor of folate to the single carbon exchange pool [14,15].

Folate status in the brain is primarily dictated by transport across the choroid plexus into the cerebrospinal fluid (CSF) where the concentration is 3–4 times higher than in the blood [16]. This transport against a concentration gradient is carried out by folate receptor alpha (FRα) expressed on the basal side of the choroid plexus epithelium, which binds MTHF and PGA with a high affinity for transport across the blood-brain barrier at physiologic folate concentrations [17]. A relative increase in blood PGA concentration could potentially decrease MTHF uptake and increase PGA uptake into the brain. The brain cannot utilize this form efficiently because of the low dihydrofolate reductase activity (DHFR) [4,5]. Therefore, using an appropriate form of folate for optimum absorption and utilization becomes critical for treating patients with cerebral folate deficiency syndrome (CFDS) or autism spectrum disorder (ASD). The accompanying autoimmune disorder that produces autoantibodies (AuAb) against FRα and blocks folate transport across the choroid plexus and thus into the brain is present in ~80% of CFDS and ~70% of ASD children [18,19]. Correcting folate deficiency in the brain requires pharmacologic doses of folate to prevent the antibodies from binding to FRα and using alternate routes such as the reduced folate carrier (RFC) for folate transport across the choroid plexus [20].

Two types of FRα autoantibodies (FRαAuAb) have been identified in a majority of children with CFDS as well as ASD: blocking AuAb that prevents the binding of folate to FRα and binding AuAb that does not [21]. These can be both IgG and IgM antibodies; in CFDS, blocking Ab with relatively high titer predominates and most of the IgG is of the IgG 4 subtype whereas relatively lower titers of binding Ab predominate in ASD, and these can be IgG2 and IgG4 subtypes as well as IgM [21]. Treatment with high dose D,L-folinic acid (Leucovorin) has proven to be beneficial in many developmental disorders including ASD, especially coexisting with FRαAuAb. Leucovorin calcium, a racemic form of folate available as a pharmaceutical grade compound, has been used at doses of 1 to 2 mg per kg body weight, up to 50 mg per day to correct CFD [19,22]. Leucovorin is a reduced form of folate that can be transported into cells via the RFC and can readily participate in folate dependent pathways without conversion to THF by the enzyme DHFR or needing a fully functional methylene tetrahydrofolate reductase (MTHFR) and methionine synthase (MS) enzymes for its utilization. Its use in cancer patients to rescue systemic toxicity from folate pathway chemotherapeutic drugs proves its efficacy in restoring folate dependent reactions [23,24]. In CFDS, it is beneficial to provide a form of folate that can readily restore CSF folate and make it available for folate pathways.

In a rat model of exposure to rat FRα specific antibodies (FRαAb) during gestation, we have shown antibody accumulation in the placenta and blocking of folate transport to the fetus [25]. Pups born to these mothers have shown learning and behavioral deficits that can be prevented with folinic acid [26]. No systematic study comparing the absorption and tissue distribution of folate forms is available. We have used young rat pups to conduct these studies to identify the form of folate best distributed to the brain in the presence of FRαAb. Although performed in rats, this study identifies a form of folate best distributed to the brain and points to potential treatment strategies best suited to treat CFD in ASD children positive for FRαAb.

## 2. Materials and Methods

### 2.1. Folate Compounds Used in the Study

Folic acid (PGA; Pteroyl glutamic acid, MP Biomedicals, Solon, OH, USA) and the 5S-methyfolate acid form (MTHF; Cerbios-Pharma SA, Bargengo, Switzerland) were suspended in water and solubilized by the dropwise addition of 0.1 N Na0H to produce the sodium salt, with D,L-folinic acid (leucovorin; Sagent, Schaumburg, CA, USA) as the calcium salt and L-folinic acid (Levofolinate; Fusilev, Spectrum Pharmaceuticals, Irvine, CA, USA) as the calcium salt. All folate forms for oral administration were diluted to a final concentration of 2 mg/mL, in 0.1 M NaPO_4_ buffer at pH 7.4. Biotin-PGA (B-PGA; Nanocs, M.W. 3400, New York, NY, USA) with the biotin PEGylated on the glutamate portion of folic acid being dissolved in water and further diluted to 25 µg in 500 µL of normal rat serum. ^3^H-PGA (specific activity 20 to 35 cpm/pg) was purchased from Moravek Biochemicals (Brea, CA, USA).

### 2.2. FRαAb and ^3^H-PGA Distribution in PND 13 Pups

The production of recombinant FRα, purification, immunization of rabbits, generation of FRαAb and characterization of these antibodies for binding and blocking FRαAb titer has been described in previous publications [21,27]. For all of the studies reported here, many different lots of antiserums from immunized rabbits were used. The IgG fraction in rabbit serum was purified on a Protein-A affinity matrix and the FRα-specific IgG fraction was determined [21,27]. The specific activity of the FRα-specific antibody varied and therefore, where applicable, the total IgG and antigen specific IgG is mentioned. Initially, the studies were performed in age group postnatal days (PND) 10–13, the youngest age we could safely inject the pups. For the FRαAb distribution studies, 4 pups were injected intraperitoneally (IP) with 1.5 mg of rabbit IgG containing 35 µg of specific anti-FRαAb or an equivalent dose of normal rabbit IgG (NRIgG) plus 50 µL normal rat serum (total volume 100 µL) on PND10, 11 and 12. The pups were euthanized 24 h post injection, the brain was collected and regions (the hippocampus, cerebral cortex, cerebellum and the midbrain) were dissected, then homogenized in 3 volumes of 0.1 M glycine/HCl, pH 2.5. The membranes were pelleted by centrifugation and the clear supernatant collected, neutralized with 1 N NaOH and the antibody concentration was measured and quantified by immunoprecipitation of ^3^H-PGA -labeled FRα antigen with anti-rat FRα and by an ELISA based assay, similar to the binding antibody determination using rat FRα immobilized in ELISA plates as the antibody capture antigen [21]. The acid release of antibody fully dissociates the antibody from the FRα antigen which remains membrane-bound and therefore, we can quantify the antibody by direct immunoprecipitation of ^3^H-PGA-FRα antigen and by standard ELISA assay to capture the antibody and quantify the IgG as previously described [21,27]. Both assays provide a quantitatively similar concentration of specific FRα IgG antibody. Additional pups were similarly injected with FRαAb (*n* = 6) or NRIgG (*n* = 6) and on PND12 they also received 5 µCi of ^3^H-PGA IP (231 ng) and were euthanized 24 h later. The brains from rats receiving ^3^H-PGA were homogenized in 3 volumes of 1 N HCl and after spinning down the membranes, a 1 mL aliquot of the clear supernatant was added to 20 mL of scintillation fluid (Ecolume, MP Biomedicals, Solon, OH, USA) to determine ^3^H-PGA in the extract. No radioactivity was left in the membrane fraction following this extraction procedure. To determine if the ^3^H radioactivity was still associated with folate, an aliquot of the extract was incubated with purified FRα which showed that all of the radioactivity could bind to the protein. The supernatant was added to a liquid scintillation cocktail for radioactivity determination in a Packard liquid scintillation counter. Handling and injecting such young pups was difficult and challenging and therefore all subsequent studies were performed in PND23 pups immediately post-weaning.

### 2.3. Brain Uptake of B-PGA in PND 23 Pups

For the time course of folate uptake into the brain, PND23 pups were injected IP with 5 µg of B-PGA (in 100 µL sterile saline + 200 µL normal rat serum), one animal was euthanized at time points ranging from 5 min to overnight, the brain was collected and placed in fixative (4% paraformaldehyde in PBS, pH 7.2) for immunohistochemical localization of B-PGA. For the paraffin-embedded brain, 5–7 µm sections were deparaffinized, equilibrated in PBS, and quenched in 3% H_2_O_2_. The accumulation of B-PGA was visualized by incubating with the avidin/biotin/peroxidase complex for 30 min (Vectastain ABC R.T.U. system, Vector Labs, Newark, CA, USA) followed by color development with 3,3′-diaminobenzidine (DAB) substrate (Vector Labs).

### 2.4. Brain Localization of FRαAb in PND 23 Pups

The pups (*n* = 2) were injected IP with 50 µg of FRαAb containing rabbit IgG (total 2.5 mg) plus 200 µL of normal rat serum in a total volume of 300 µL. The pups were euthanized and their brains were processed for immunohistochemical localization of FRαAb as described above. Following blocking with NGS, tissue sections were first incubated with biotinylated goat anti rabbit IgG 100 µL (1:500 dilution) for 30 min (Vector labs, Newark, CA, USA) followed by incubating with ABC reagent for 30 min and color development using DAB substrate. Another set of pups (*n* = 2) was similarly injected with NRIgG as a control and processed by the same immunohistochemical procedure, to serve as a negative control.

### 2.5. Absorption and Tissue Distribution of ^3^H-PGA in PND23 Pups

The time course of PGA absorption in pups was determined by orally administering 5 µCi of ^3^H-PGA (231 ng) in 175 µL of water and euthanizing the pups at 30, 60, 120, 240 and 360 min. Tissues were collected, and ^3^H-PGA was determined as described above. To study the effect of FRαAb on ^3^H-PGA distribution, the pups were first injected IP with 50 µg FRαAb specific antibody as described above followed by another 50 µg 16h later. This was done to ensure that an adequate amount of antibody was around, both membrane-bound and in circulation. Two hours after the second FRαAb injection, the pups received 5 µCi of ^3^H^−^PGA IP and were euthanized 6h later. A group of pups similarly injected with non-immune NRIgG served as controls. The primary objective of these studies was to quantify the distribution of PGA in the young rat brain and to determine if this distribution was affected by FRαAb. For this analysis, the brain was dissected into the cerebrum, hippocampus, cerebellum and subcortex (comprised of thalamic and hypothalamic nuclei, basal ganglia, amygdala, and midbrain). The tissue was homogenized in 3 volumes of 0.1 N HCl, the membranes were separated by centrifugation (14,000× *g* for 10 min) and radioactivity in the clear supernatant was determined as described above.

### 2.6. Dosing of PND23 Rats with Folate Compounds

For the absorption and brain uptake of folate forms, PND23 male rat pups weighing approximately 50 g were deprived of food overnight and administered folate forms orally at a dose of 4 mg/kg (in 175 µL of water). There were in total 9 groups of pups that had 3 pups within in each group. The first group was not given any folate form or antibody and was treated as a baseline control for endogenous folate (AA). The remaining 8 groups were split into antibody groups where they received either NRIgG (AB-AE) or FRαAb (ABb-AEb) prior to their folate form administration. The 4 groups within each antibody group were separated to be administered folic acid (PGA; AB, ABb), L-methylfolate (MTHF; AC, ACb), D,L-folinic acid (Leucovorin (D,L-Fol); AD, ADb) and levofolinate (L-folinic acid (L-Fol); AE, AEb). The rabbit polyclonal IgG fraction (5 mg) administered IP contained 100 µg of specific anti rat FRαAb plus 200 µL of rat serum (300 µL total volume) 24 h prior to oral dosing with folate forms to determine if FRαAb would affect tissue uptake of folates at the pharmacologic dose administered and to identify the form of folate most effective in restoring brain uptake in the presence of FRαAb. The animals were euthanized 24 h after oral dosing of the compound, by CO_2_ asphyxiation, the tissues were collected, briefly rinsed in ice-cold saline, suspended in 3 volumes of 0.1 M NaPO_4_ buffer containing 2% ascorbic acid adjusted to pH 7.4, homogenized using a polytron and kept frozen at −20 °C. To measure MTHF concentration, an aliquot of the homogenate was diluted in 2% ascorbic acid containing phosphate buffer (1:5 to 1:10 dilution), in a glass tube, sealed with parafilm and foil, and placed in a boiling water bath for 10 min. The sample was cooled and centrifuged at 3000× *g* for 5 min. The clear supernatant was transferred to another tube and assayed for MTHF concentration by a sequential binding assay using purified bovine FRα as the binding protein, ^3^H-PGA as the tracer and freshly prepared MTHF as the standard. The assay as performed is specific for MTHF in tissue extracts and the extraction procedure used yields a quantitative recovery of tissue MTHF with negligible interference from other intracellular forms of folate at the concentrations present in tissues [28,29].

All rats used in these studies had free access to water and food with a normal diet containing 2 mg folic acid per kg chow as recommended by the American Institute of Nutrition (1977) but were deprived of food overnight prior to their use in the studies specified. The rats were maintained at 22 °C and on a 12 h light/dark cycle and the experimental protocols were approved by the Animal Care and Use Committee of the State University of New York, Downstate Medical Center, Brooklyn, NY, USA.

### 2.7. Statistical Analysis

To determine the differences in folate uptake between FRαAb- and NRIgG-injected PND13 pups, a one-way ANOVA was completed using SPSS (IBM, Armonk, NY, USA). A *p*-value of less than 0.05 was considered significant.

To determine which folate form given at PND23 was most efficiently transported into tissues, jamovi software (https://www.jamovi.org (accessed on 1 July 2022), Sydney, Australia) was used. A one-way ANOVA and Welch’s test were conducted to compare the accumulation of MTHF in the PND23 liver, kidney, cerebrum, and cerebellum. A post-hoc Tukey’s test compared the significant differences between the folate form with either NRIgG (AB-AE) or FRαAb (ABb-AEb) present and values where no folate or antibody was given (endogenous/baseline: AA). All means, standard deviations, and asterisks representing *p*-values of the comparisons previously described are shown in Table 1. To compare the difference of folate uptake in the presence of either NRIgG or FRαAb, a one-way ANOVA was conducted with a post-hoc Tukey’s test or Games-Howell test depending on verification from the Shapiro–Wilk test for normality. The *p*-values comparing NRIgG and FRαAb administered groups are reported in Supplementary Table S1.

## 3. Results

### 3.1. Distribution of FRαAb and Blocking of Folate Uptake in PND 13 Rat Brain

**Figure 1:**
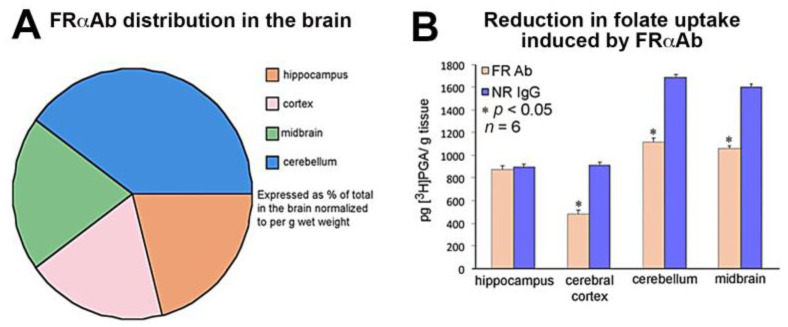
Anti-folate receptor antibody distribution and its effect on folate uptake in PND13 rat brain. (**A**). Antibody distribution in the cortex, midbrain, and hippocampus and in the cerebellum (total recovered was 1080 pg IgG antibody/g of whole brain tissue). The pie chart shows % distribution per gram of tissue (*n* = 4); (**B**). Uptake of ^3^H-PGA in these rats showed a 30–40% decrease in uptake in the cortex, midbrain, and cerebellum (*n* = 6 for both FRαAb and NR IgG groups). Asterisk (*) represents one-way ANOVA statistical significance between FRαAb and NRIgG across each brain region (*p* < 0.05).

The FRαAb extracted from dissected regions of the brain and quantified, yielded a total of 1080 pg IgG protein/g of brain tissue (wet weight), 24 h after the last dose. Of this, about 40% was localized to the cerebellum and the remaining 60% was equally distributed in the cortex, midbrain, and the hippocampus (Figure 1A). This antibody distribution affected ^3^H-PGA uptake into the brain as seen by a 30% decrease in radioactivity in the cortex, cerebellum, and midbrain but not in the hippocampus, even though it had the same amount of antibody as the cerebral cortex and midbrain (Figure 1B).

### 3.2. Biotin-PGA (B-PGA) Uptake and Distribution in PND 23 Brain

**Figure 2:**
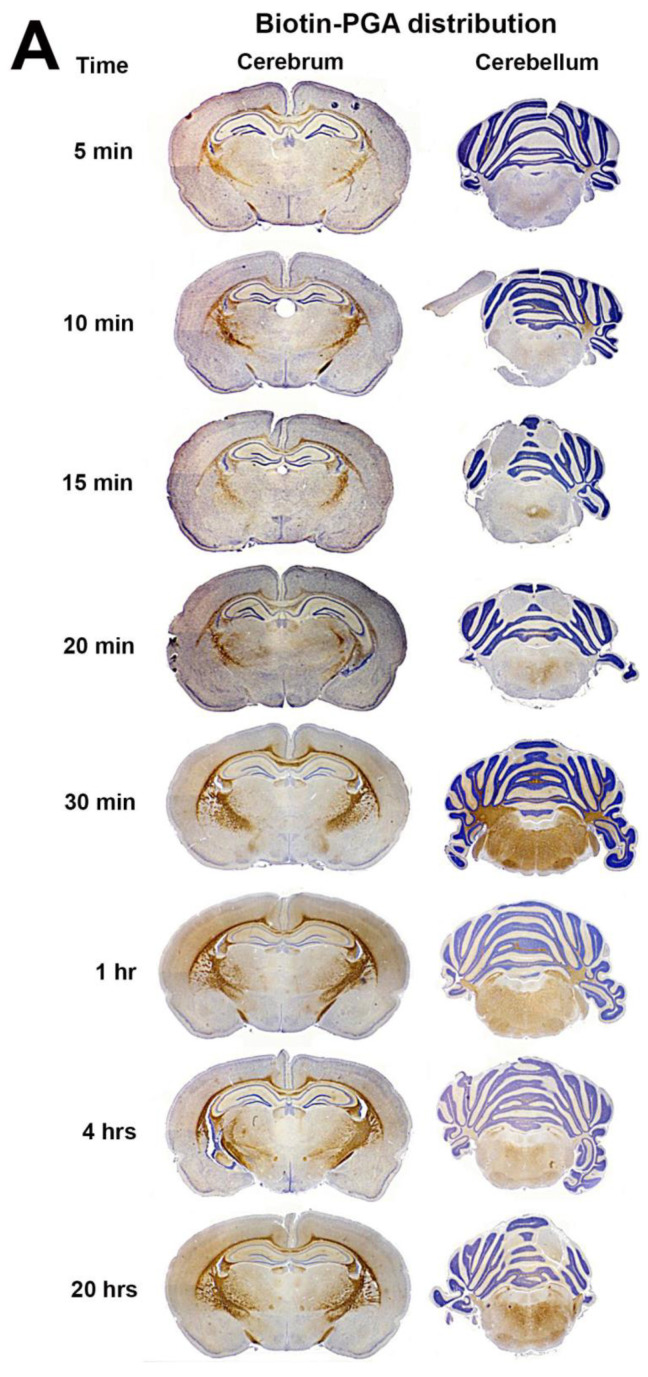

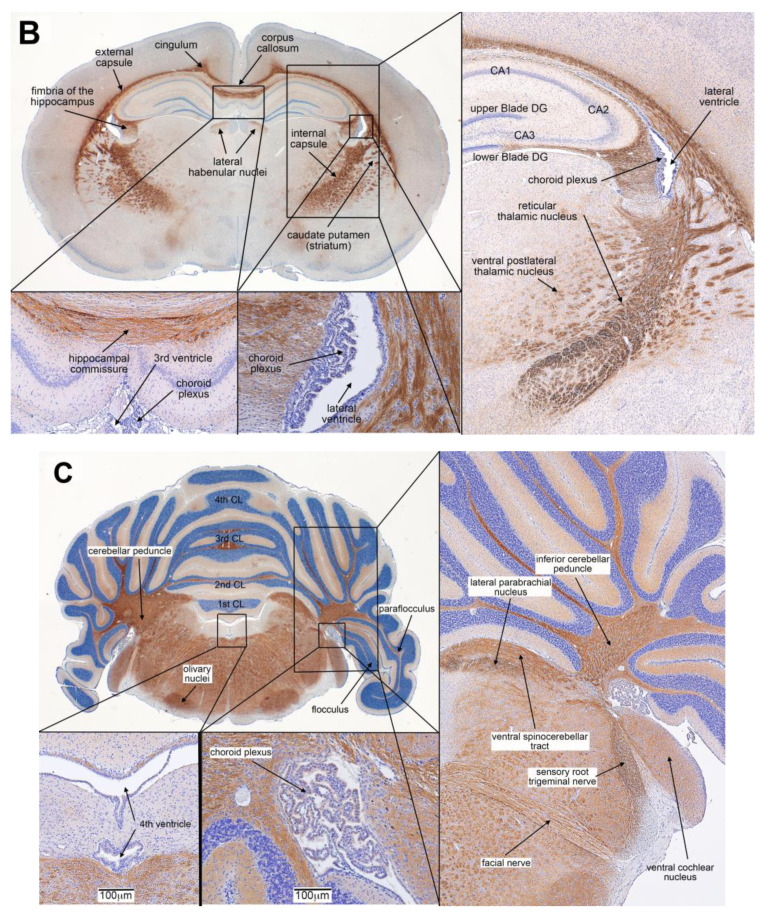
(**A**). Time course of Biotin-PGA uptake and distribution in the PND23 rat brain, immunostained for B-PGA (brown) and hematoxylin (blue/purple). (**B**). Regional distribution of B-PGA in the PND23 cerebrum at 30 min. Extensive accumulation of folate is seen in the white matter tracts, immunostained for B-PGA and hematoxylin. (Abbreviations: CA1; cornu ammonis 1, CA2; cornu ammonis 2, CA3; cornu ammonis 3, DG; dentate gyrus.). (**C**). Regional distribution of B-PGA in the PND23 cerebellum at 30 min, immunostained for B-PGA and hematoxylin. (Abbreviation: CL; Cerebellar lobule).

While orally administered folates take 30–60 min to reach peak folate concentration in the blood, the IP administered B-PGA appears rapidly in the circulation with transport to tissues including the brain. B-PGA is detected in the cerebrum within 5 min of IP administration, mostly in the white matter tracts. This accumulation appears to peak around 60 min, with little or no loss of folate distribution overnight (20 h). The appearance of B-PGA in the cerebellum is slightly delayed with some folate detected at 15 min. Substantial accumulation is seen at 30 min with some decrease overnight (Figure 2A). Regional distribution of B-PGA was evaluated in a coronal section of a 30 min brain cortex (Figure 2B). Evaluation of the choroid plexus at all time points including the 30 min brain showed very little B-PGA in the choroid plexus. In the cerebrum, folate was concentrated along the white matter tracts with extensive accumulation in the corpus callosum, fimbriae, internal and external capsular regions, within the cells. Similar evaluation of 30 min cerebellum also showed extensive folate accumulation in the white matter tracts. Because of the greater uptake and accumulation, some folate can be seen in the choroid plexus (Figure 2C). Maximum B-PGA accumulation in the cerebellum was seen at 30 min with some decrease overnight (20 h).

To determine if we were indeed visualizing B-PGA and not free biotin cleaved from B-PGA, we conducted additional experiments with biotin and B-PGA. An identical dose of injected biotin distributed differently even though some overlap in regional distribution was seen, and a 500-fold molar excess of folic acid did not decrease this distribution. On the other hand, a 500-fold excess of folic acid administered along with B-PGA showed a clear decrease in the distribution of B-PGA (see Supplementary Figure S1).

### 3.3. Distribution of IP Injected FRαAb in the Brain

**Figure 3:**
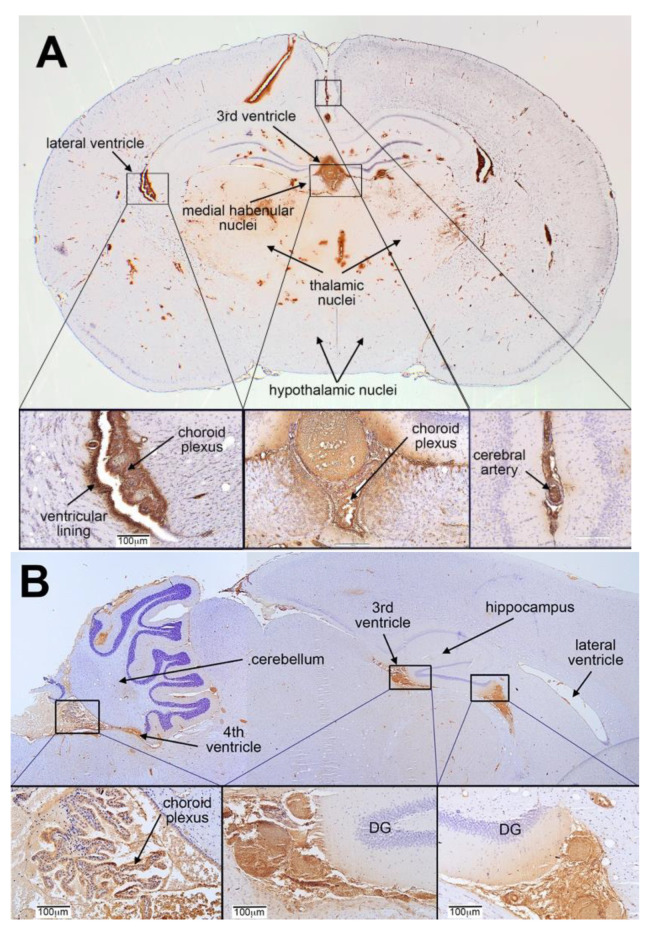
(**A**). Localization of FRαAb in the PND23 cerebrum. Coronal section shows FRαAb distribution within the blood vessels, microvasculature and the choroid plexus within the ventricles. (**B**). Localization of FRαAb in the PND23 cerebellum. Sagittal section of the brain shows FRαAb distribution in the blood vessels and in the vasculature of the 3rd and 4th ventricles including the choroid plexus. (Abbreviation: DG = dentate gyrus).

Direct visualization of FRαAb in histologic sections of the brain showed the antibody localizing to the blood vessels and the microvasculature throughout the brain as seen in the coronal section of the cerebrum (Figure 3A). Closer examination showed accumulation of the antibody in the choroid plexus and both large and small blood vessels throughout the brain parenchyma with very little or none outside the vasculature (Figure 3A). Similar distribution of FRαAb was seen in the cerebellum as shown in a sagittal section of the rat brain (Figure 3B). In contrast, NRIgG-injected animals do not show the same distribution (see Supplementary Figure S2).

### 3.4. Time Course of Folate Absorption, Tissue Distribution and the Effect of FRαAb on Distribution in the Brain

**Figure 4:**
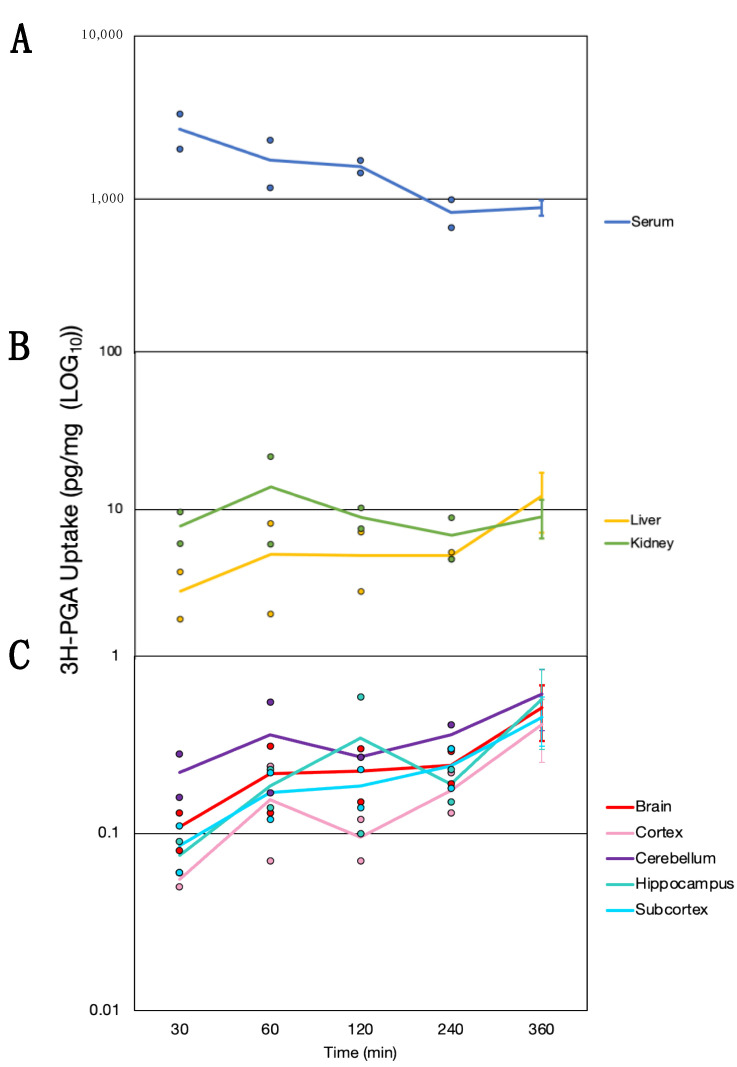
The time course of orally administered ^3^H-PGA appearing in (**A**) blood serum (blue), (**B**) liver (yellow) and kidney (green), and (**C**) brain regions (total Brain (red), cortex (pink), cerebellum (purple), hippocampus (turquoise), subcortex (blue)) over time. Sample sizes; *n* = 2 for 30–240 min *n* = 6 for 360 min. Solid lines represent the mean while individual values are shown for timepoints 30–240 min. The standard deviation is shown for 360 min.

This low-dose oral administration of ^3^H-PGA was given to determine how the gut handles the absorption and tissue distribution of the daily dietary intake of folate. Serum concentration was highest at 30 min and decreased over the next 3.5 h reaching a plateau between 240 and 360 min (Figure 4A). There was a rapid increase in the kidneys between 30 and 60 min followed by an initial decline and then an increase at 360 min. The liver showed a slow increase with highest accumulation at 360 min (Figure 4B). Uptake was similar in all regions of the brain with PGA continuing to accumulate at 360 min (Figure 4C).

**Figure 5:**
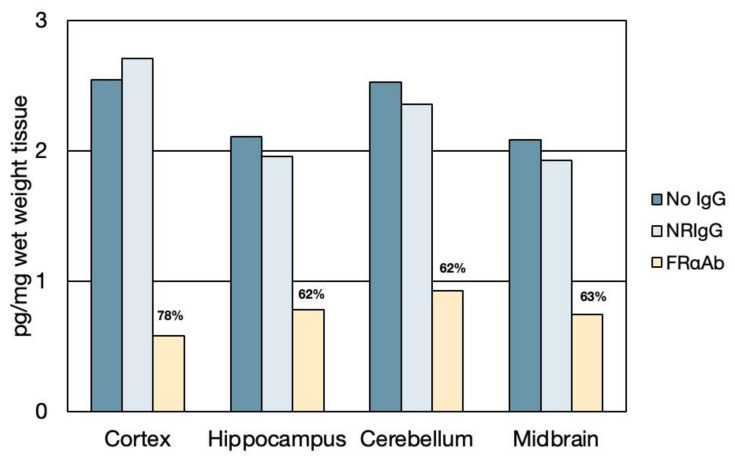
Effect of FRαAb on brain uptake of ^3^H-PGA. While NRIgG had no effect on ^3^H-PGA uptake, a decrease of more than 60% was seen in all regions of the brain of the FRαAb-injected animal (a single animal was used for each profile).

Tissue distribution of IP-injected ^3^H-PGA (231 ng) was similar to the oral dosing, with the maximum amount retained in the liver and kidney, respectively (data not shown). The brain uptake accounted for a relatively small amount of the dose with more uptake in the cerebellum compared to the cerebrum. This distribution was altered by antibody to FRαAb with a 60–70% decrease in various regions of the brain. This decrease in uptake was seen only in FRαAb-injected rats and not in the NRIgG-injected rat (Figure 5).

### 3.5. Tissue Distribution of Methylfolate Post Oral Dosing with Folate Forms

In control animals on a normal folate replete rat chow, the liver has about 2.5-fold higher folate than the kidneys. This ratio is decreased when a single pharmacologic dose is administered indicating increased shunting of folates to the kidneys for excretion. Concomitantly, an increase is seen in liver folate with all four forms of folate administered with a further increase in rats administered FRαAb with the highest increase with MTHF (AA to AB (*p* = 0.312); AA to ABb (*p* < 0.001); AA to AC (*p* = 0.002); AA to ACb (*p* = 0.005); AA to AD (*p* = 0.018); AA to ADb (*p* = 0.735); AA to AE (*p* = 0.047); AA to AEb (*p* = 0.173)). On average, the distribution of MTHF in the liver and kidneys was similar in all forms of folate and was not greatly altered by the presence or absence of FRαAb. However, the kidneys had almost double the amount of MTHF compared to the baseline value (AA) ((AA to AB (*p* = 0.225); AA to ABb (*p* = 0.118); AA to AC (*p* < 0.001); AA to ACb (*p* < 0.001); AA to AD (*p* = 0.427); AA to ADb (*p* = 0.060); AA to AE (*p* = 0.406); AA to AEb (*p* = 0.394)) (Table 1).

In the brain, all folate forms were better incorporated into the cerebellum compared to the cerebrum. However, both D,L-folinic acid (Leucovorin) and levofolinate were better transported into the brain in the presence or absence of FRαAb at the dose administered. The distribution of the two folate forms was 4–5 times higher in the cerebellum compared to the cerebrum (AA to AB (cerebellum *p*-value = 0.194, cerebrum *p*-value = 0.163); AA to ABb (cerebellum *p*-value = 0.020, cerebrum *p*-value < 0.001); AA to AC (cerebellum *p*-value = 0.109, cerebrum *p*-value = 0.549); AA to ACb (cerebellum *p*-value < 0.001, cerebrum *p*-value = 0.58); AA to AD (cerebellum *p*-value = 0.003, cerebrum *p*-value < 0.001); AA to ADb (cerebellum *p*-value = 0.066, cerebrum *p*-value = 0.056); AA to AE (cerebellum *p*-value < 0.001, cerebrum *p*-value = 0.005); AA to AEb (cerebellum *p*-value < 0.001, cerebrum *p*-value = 0.005)) (Table 1). Both tissues accumulated twice as much folate from Leucovorin and levofolinate compared to PGA and MTHF (AD to AE (cerebellum *p*-value = 0.032, cerebrum *p*-value = 0.071); ADb to AEb (cerebellum *p*-value = 0.999, cerebrum *p*-value = 1); AD to AB (cerebellum *p*-value = 0.99, cerebrum *p*-value = 0.307); ADb to ABb (cerebellum *p*-value = 0.006, cerebrum *p*-value = 0.048); AD to AC (cerebellum *p*-value = 0.62, cerebrum *p*-value = 0.021); ADb to ACb (cerebellum *p*-value = 0.886, cerebrum *p*-value < 0.001); AE to AB (cerebellum *p*-value = 0.007, cerebrum *p*-value < 0.001); AEb to ABb (cerebellum *p*-value = 0.018, cerebrum *p*-value = 0.084); AE to AC (cerebellum *p*-value < 0.001, cerebrum *p*-value < 0.001); AEb to ACb(cerebellum *p*-value = 0.995, cerebrum *p*-value < 0.001); AB to AC (cerebellum *p*-value = 0.966, cerebrum *p*-value = 0.735); ABb to ACb (cerebellum *p*-value = 0.07, cerebrum *p*-value = 0.133)) (Supplemental Table S1). In terms of overall concentration of tissue MTHF 24 h post dosing, the liver/kidney ratio did not alter much while there was a substantial increase in the cerebellum/cerebrum ratio indicating an even greater retention of MTHF in the cerebellum.

## 4. Discussion

Distribution of FRαAb in the rat brain and its effect on ^3^H-PGA uptake is evident both in PND13 and PND23 brains. FRαAb does not appear to reach the cellular structures of the brain parenchyma but accumulates in the choroid plexus, blood vessels and the microvasculature and blocks folate transport to the brain as indicated by a decreased uptake of folate in various regions. The lack of effect in the hippocampus of the very young pups is puzzling, since the region contained adequate antibody. This is not seen in older pups and may be related to the structural development, blood supply and folate transport to this specific region in the PND13 brain.

Rapid uptake of IP-injected B-PGA in the brain may be attributed to the rapid blood flow to the brain, which is estimated to be about 1 mL/g tissue/min [30]. B-PGA accumulation is seen exclusively in the white matter, specifically in areas controlling pathways involved in memory consolidation and impulse transmission. This localization predominately in the white matter and not in grey matter may be related to the fact that in the early development of humans, glial cell proliferation, particularly oligodendrocytes, rapidly increases during the first three years [31]. In the cortex, the fimbria-fornix area is associated with spatial memory [32]. White matter pathways surrounding the hippocampus are crucial to its function because they contain the afferent and efferent connections associated with memory and emotional processing. The fornix connects various nodes of limbic circuitry and plays a role in cognition and episodic memory recall [33]. The internal capsule functions as a communication pathway between areas of the cerebral cortex [34]. The external capsule comprises cholinergic fibers from the basal forebrain to the cerebral cortex and joins the internal capsule around the lentiform nucleus [35]. The dentate gyrus collects sensory inputs and forms an unique locus of memory and stimuli to play an important role in learning and memory [36]. The detection of folate in these regions suggests a role for this vitamin in many of the specialized functions of cells and connections in these regions, as evidenced by disruptions in folate metabolism leading to molecular, structural, and behavioral impairments [37]. Evidence of hypomyelination of white matter tracts in folate deficiency and genetic cases of CFD also suggest that folate is required for the development of these regions [38].

The cerebellum, because of its greater blood flow [39], after an initial lag, accumulates more folate in the first 30 min, but also appears to lose some folate as the blood is depleted of folate with time. This may be due to redistribution of the initial uptake. However, at a steady state, the cerebellum accumulates more folate than the cerebrum. On a unit-weight basis more folate is cycled through the cerebellum than the cerebrum and folate depletion may affect many of its functions. While the cerebellum is known to be involved in regulating movements and consolidating working memory, its role in emotion, impulsive response, attention, and memory is also beginning to emerge [40]. The small number of animals used in each of the experiments and the single rat used to show blocking of radioactive folate into the brain do not provide for a rigorous evaluation of statistical significance and biological variations and therefore, the study should be considered as a pilot observation.

Many mechanisms exist for folate uptake into different organs and tissues as defined from rodent and ex vivo models. In the upper gut, transport across the ileal enterocyte is carried out by the proton-coupled folate transporter (PCFT) [41]. Absorbed folate is rapidly converted to MTHF by the folate pathway enzymes and therefore at low concentrations only MTHF would appear in the portal blood, which is first shunted to the liver. At this point, a process must exist to increase the local MTHF concentration because MTHF is taken up by hepatocytes primarily via the RFC, which is a low-affinity, high-capacity transporter for MTHF storage in the liver. When a large dose is administered, substantial amounts of unconverted folate form would appear in the blood and potentially both unconverted and the form converted to MTHF would be available for tissue uptake, including the brain [25]. Excess folate is rapidly excreted primarily via the kidneys. The kidneys are a second storage site, and a substantial amount of absorbed folate is directed to the kidneys where it is stored as well as excreted in large quantities when a high dose is administered. This excretion allows for rapid disposal of excess folate and normalization of tissue and blood folate status within a 24 h period. At physiologic dosing, most of the folate directed to the kidneys is captured by FRα expressed in the renal tubules and is reabsorbed [42]. Under normal conditions, this would be MTHF. However, if there is some PGA, the FRα expressed on the renal tubules would capture this because of its higher affinity compared to MTHF, with a resultant loss of MTHF in the urine.

Epithelial cells in all tissues throughout the body express FRα, which has high affinity for PGA and MTHF and, therefore, is the primary receptor for physiologic cellular uptake. Folate delivery to the brain is a unique process whereby transport must occur across the blood-brain barrier. Even though RFC and PCFT are expressed in the choroid plexus cells, it is the high affinity FRα, expressed on the basal side of the choroid plexus epithelium, that operates as the primary transporter of folate into the CSF and the brain. Vesicular transport via exosomes has been reported as a potential mechanism by which FRα-bound folate is transported into the CSF and ultimately into the brain [43]. However, the exact mechanism by which FRα-bound folate is released into the CSF and how this passes the ependymal cells and enters the parenchyma of the brain is still not clear. Extensive characterization of the folate transport mechanism across the choroid plexus has suggested active transport akin to the transport of amino acids with potential roles for PCFT and RFC in releasing it into the CSF and uptake into the brain [17,44,45]. Exosome shuttling seems to be an interesting mechanism proposed but needs further scrutiny [43].

**Figure 6:**
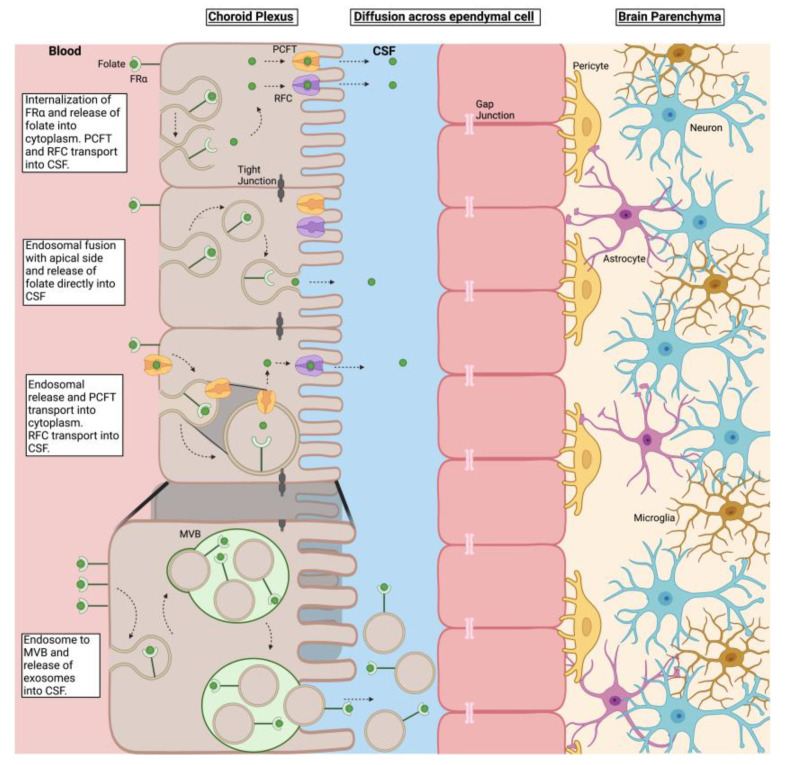
Potential mechanism of folate uptake into the choroid plexus epithelium and transport into the CSF. This active transport results in a 3–4-fold concentration of folate in the CSF compared to plasma (Abbreviations: cerebral spinal fluid; CSF, folate receptor alpha; FRα, multivesicular body; MVB, proton-coupled folate transporter; PCFT, reduced folate carrier; RFC).

Based on staining for folate transporters in the CP, and published data, the following mechanisms may operate to transport folate across the blood-brain barrier into the CSF as illustrated in Figure 6. 1. FRα with folate bound could be taken up by endocytosis and released into the cytoplasm where the concentration could rise to make the RFC operate and transport folate into CSF. A similar mechanism could also work for PCFT. 2. An endosome could translocate to the apical side and release folate into the CSF, i.e., vesicular translocation of FRα from the basal to the apical side with the cargo. In this case, folate being discharged into the CSF could also explain the intense staining for FRα on the apical side. 3. An endosome could form with PCFT, the folate could be released and transported into the cytoplasm by the endosome-associated PCFT and the RFC could transport the free folate into the CSF. 4. An endosome could form an exosome and carry FRα with the bound folate into the CSF and then go across the ependymal cells to deliver the folate to neuronal cells. Even though it has been experimentally shown in a mouse model and rat choroid plexus cells transfected to express human FRα [43], this mechanism operating in vivo has not been confirmed. If indeed exosomes are involved, one would expect to see staining for FRα in the CSF compartment, which we do not see. We have also failed to detect any FRα in the CSF of patients (data not shown). The 3–4-fold higher concentration of folate in the CSF compared to the plasma appears to be free folate not bound to any protein or vesicle. Earlier-published evidence points to a basal FRα dependent uptake and RFC and/or PCFT involved transport into the CSF [17,46]. This could potentially lead to a high accumulation of folate in the CSF. Once a high CSF concentration is achieved, transport across the ependymal cell layer and into the brain parenchyma can be explained by simple diffusion and uptake into brain cells by the RFC. These speculative mechanisms need further evaluation.

Pharmacologic dosing with folate can restore the low CSF folate in CFDS and ASD and therefore, both PCFT and RFC may play a role in this process [47]. Restoration of brain folate with D,L-folinic acid treatment produces significant clinical improvement, especially in language, communication, and social interaction [48]. Based on available data on clinical response, the use of D,L-folinic acid as a first line of treatment is likely to increase the need for this form of intervention and therefore it is necessary to identify the form of folate most effective in restoring brain folate.

A comparative study of the absorption and tissue distribution of folate forms was undertaken to identify the most suitable form of folate for the treatment of ASD and CFDS because this treatment would require long-term use of the compound at relatively high doses. Since the treatment is aimed at overcoming the blocking effects of FRαAb on folate transport to the brain, we evaluated uptake by measuring MTHF concentration in the brain and the effect of FRαAb on this process after administering each compound orally. At the dose tested, all folate compounds increased tissue levels of MTHF, however, levofolinate appeared to be better than the other forms, especially in the brain. Based on the localization of B-PGA, uptake of IP-injected folate is rapid with the appearance of folate in the cerebrum within 5 min. Once in the blood, folate is rapidly distributed to the brain and appears to be mostly retained in the white matter.

Folates as major players in DNA/RNA synthesis and intermediary metabolism, are essential throughout life but more so during fetal and neonatal periods, for normal brain development and function. Because of their role in methylation reactions, epigenetic control of gene expression and synthesis of amino acids and neurotransmitters, folate deficiency or disruption of folate metabolism can have multiple deleterious effects on brain development and function [49]. This is evident from NTD pregnancy and developmental disorders, such as CFDS and ASD, connected to inadequate folate transport to the fetus and to the brain [50]. Other than potential dietary folate deficiency, most of these conditions are associated with AuAb against FRα. These antibodies can block folate transport across the placenta to the fetus and across the choroid plexus, to the brain [50]. Local inflammation triggered by antigen/antibody interaction is another contributing factor to the pathology as evidenced from the rat model [26]. In CFDS and ASD, pharmacologic dosing with D,L-folinic acid has restored the low CSF folate and improved brain function [18,22,51]. While folic acid is an inexpensive and stable form of folate is used in food fortification and vitamin pills, it may not be suitable for use in the treatment of CFDS and ASD where large daily doses are needed to overcome the effects of FRαAb and to transport adequate folate to the brain by alternate routes such as the RFC. In addition, recent studies have suggested that unmetabolized folic acid accumulates in the blood and tissues and could have a deleterious effect on folate metabolism [52]. If this is proven to be true, then pharmacologic dosing with folic acid would be contraindicated. MTHF is another form of folate that may be used but first-pass metabolism of this compound requires a fully functional MTHFR and MS enzymes and cobalamin-replete status for utilizing this form of folate. The stability of MTHF is another concern that needs addressing before its wider use. Leucovorin, also known as D,L-folinic acid (D,L-N^5^ formyl THF), is a reduced form of folate that can be metabolized to methylene-THF and utilized in folate pathways. It is relatively stable as a Ca^2+^ or Na^+^ salt and has been used in large doses for more than a decade to rescue patients from the toxic effects of chemotherapy and more recently to treat CFDS and ASD [23,24,48,51]. Since the compound is a racemic mixture and only the levo (S) form is active, there is some concern about the dextro (R) form accumulating and interfering with folate utilization [53,54]. The recent availability of levofolinate has made this the compound of choice since the dose can be reduced by 50% and the compound is considered stable, both as a liquid formulation and solid form. The availability of liquid formulation makes its use easy even for younger children and infants.

## 5. Conclusions

Circulating folate is rapidly transported to the brain and accumulates in the white matter tracts in young rat brains. FRαAb is also rapidly transported and avidly collects in the choroid plexus and vasculature. In the presence of FRαAb, folate uptake into the brain is substantially decreased. High-dose folate forms can restore brain folate. Levofolinate appears to be best suited for restoring brain folate in the presence of FRαAb.

## Figures and Tables

**Table 1:** Tissue concentration of methylfolate following orally administered folate forms 24 h post dosing and the effect of FRαAb on this distribution in PND23 rat pups (*n* = 3).

Code	Condition	Methylfolate (ng/mg Wet Weight)					
		Mean ± SD	Mean ± SD	Ratio	Mean ± SD	Mean ± SD	Ratio
		Liver	Kidney	Liver	Cerebellum	Cerebrum	Cerebellum
				Kidney			Cerebrum
**AA**	**Control**	125 ± 5	46 ± 3	3	11 ± 4	4 ± 0.5	3
**AB**	**PGA + NRIgG**	150 ± 33	74 ± 28	2	38 ± 25	7 ± 2	6
**ABb**	**PGA + FRαAb**	160 ± 5 ***	67 ± 18	2	21 ± 2 *	8 ± 0.5 ***	3
**AC**	**MTHF + NRIgG**	159 ± 6 **	87 ± 6 ***	2	26 ± 10	4 ± 0.2	6
**ACb**	**MTHF + FRαAb**	250 ± 18 **	116 ± 13 ***	2	61 ± 1 ***	4 ± 0.5	16
**AD**	**Leucovorin + NRIgG**	92 ± 10 **	51 ± 8	2	48 ± 7 **	10 ± 0.5 ***	5
**ADb**	**Leucovorin + FRαAb**	134 ± 41	60 ± 9	3	77 ± 31	14 ± 4	6
**AE**	**Levofolinate + NRIgG**	80 ± 19 *	52 ± 11	2	93 ± 6 ***	15 ± 2 **	6
**AEb**	**Levofolinate + FRαAb**	133 ± 8	49 ± 4	3	70 ± 2 ***	13 ± 2 **	5

Asterisks refer to the ANOVA, post-hoc Tukey’s test *p*-values that compared the folate forms administered in the presence of either NRIgG (PGA; AB, MTHF; AC, Leucovorin (D,L-Fol); AD, Levofolinate (L-Fol); AE) or FRαAb (PGA; ABb, MTHF; ACb, Leucovorin (D,L-Fol); ADb, Levofolinate (L-Fol); AEb) to endogenous/baseline folate (control; AA); * *p* < 0.05, ** *p* < 0.01 *** *p* < 0.001.

## Data Availability

Not applicable.

## Supplementary Materials

The following supporting information can be downloaded at: https://www.mdpi.com/article/10.3390/nu15051167/s1, Figure S1: Localization of BPGA, BPGA with 500-fold unlabeled PGA, Biotin, and Biotin with 500-fold unlabeled PGA; Figure S2: Localization of NRIgG and FRαAb in PND23 Table S1: Summary of Statistical Analysis for Table 1.
