# Supplementary material for: Brain Uptake of Folate Forms in the Presence of Folate Receptor Alpha Antibodies in Young Rats: Folate and Antibody Distribution
Source: Nutrients. 2023 Feb 25;15(5):1167. doi: 10.3390/nu15051167 (PMC10005127; doi:10.3390/nu15051167)
Supplement: Supplementary file 1 [file nutrients-15-01167-s001.zip › nutrients-2215803-supplementary.pdf]

Code	Tissue	AB	ABb	AC	ACb	AD	ADb	AE	AEb
AB	Liver	-	0.999	0.999	**<0.001**	0.066	0.981	**0.018**	0.978
	Kidney	-	0.998	0.936	**0.028**	0.496	0.933	0.581	0.408
	Cerebrum	-	0.951	0.785	0.607	0.307	**0.006**	**<0.001**	**0.011**
	Cerebellum	-	0.821	0.966	0.61	0.990	0.092	**0.007**	0.242
ABb	Liver	-	-	1	**0.002**	**0.021**	0.796	**0.006**	0.787
	Kidney	-	-	0.663	**0.009**	0.83	0.999	0.891	0.748
	Cerebrum	-	-	0.222	0.133	0.882	**0.048**	**0.006**	0.084
	Cerebellum	-	-	1	0.07	0.371	**0.006**	**<0.001**	**0.018**
AC	Liver	-	-	-	**0.002**	**0.024**	0.824	**0.006**	0.815
	Kidney	-	-	-	0.218	0.085	0.348	0.110	0.064
	Cerebrum	-	-	-	1	**0.021**	**<0.001**	**<0.001**	**<0.001**
	Cerebellum	-	-	-	0.152	0.62	**0.014**	**<0.001**	**0.041**
ACb	Liver	-	-	-	-	**<0.001**	**<0.001**	**<0.001**	**<0.001**
	Kidney	-	-	-	-	**<0.001**	**0.003**	**<0.001**	**<0.001**
	Cerebrum	-	-	-	-	**0.011**	**<0.001**	**<0.001**	**<0.001**
	Cerebellum	-	-	-	-	0.963	0.886	0.202	0.995
AD	Liver	-	-	-	-	-	0.302	0.997	0.31
	Kidney	-	-	-	-	-	0.985	1	1
	Cerebrum	-	-	-	-	-	0.412	0.071	0.586
	Cerebellum	-	-	-	-	-	0.337	**0.032**	0.66
ADb	Liver	-	-	-	-	-	-	0.1	1
	Kidney	-	-	-	-	-	-	0.995	0.963
	Cerebrum	-	-	-	-	-	-	0.948	1
	Cerebellum	-	-	-	-	-	-	0.858	0.999
AE	Liver	-	-	-	-	-	-	-	0.103
	Kidney	-	-	-	-	-	-	-	1
	Cerebrum	-	-	-	-	-	-	-	0.843
	Cerebellum	-	-	-	-	-	-	-	0.541

Supplementary Table 1: Summary of Statistical Analysis for Table 1. ANOVA post-hoc Tukey's test p-values of each folate form given and Ab injected interactions in liver, kidney, cerebrum, and cerebellum (n=3 for each group). Both d,l-folinic acid (Leucovorin) and levofolinate, regardless of either antibody injected, have significantly increased MTHF uptake in brain (cerebrum and cerebellum) and significantly decreased MTHF uptake in liver and kidney (significant p-values are shown in **bold**) (Abbreviations (Code; antibody injected/folate form orally given): **AB**; NRIgG/PGA, **ABb**; FRαAb/PGA, **AC**; NRIgG/5MTHF, **ACb**; FRαAb/5MTHF, **AD**; NRIgG/Leucovorin, **ADb**; FRαAb/Leucovorin, **AE**; NRIgG/Levofolinate, **AEb**; FRαAb/Levofolinate)

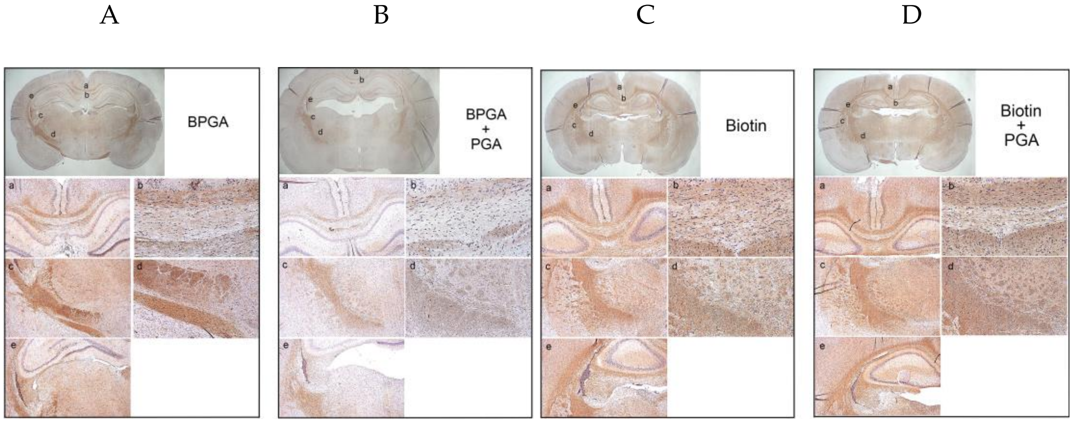

Supplementary Figure S1: Localization of B-PGA (A), B-PGA with 500-fold unlabeled PGA (B), Biotin (C), Biotin with 500-fold unlabeled PGA (D). Uptake and distribution of B-PGA in select regions of the rat brain is confirmed by decreased distribution in the presence of a 500-fold excess folic acid. Even though biotin is distributed to similar regions and overlaps with B-PGA, folic acid does not affect this distribution, indicating distinct uptake and distribution of the two compounds in the brain. The distribution of B-PGA and biotin overlaps in white matter tracts which include the corpus callosum, cingulum, hippocampal commissure, fornix, and internal capsule. These regions are associated with the pathways of the limbic system, the historically relevant regions of emotion and memory processing. From top to bottom in subpanels a and b in all panels of the figure (A-D) represent the cingulum, corpus callosum, and hippocampal commissure in that order. The cingulum is a bundle of axons which connects the cingulate gyrus and entorhinal cortex. This region is associated with cognitive control such as working memory and emotion processing. Damage to this region has been shown to lead to mild cognitive impairment which also damages the hippocampus because the connections to processing information is disrupted. Both the corpus callosum and the hippocampal commissure as their function connect the right and left hemispheres and hippocampi respectively. Subpanels c and d show the internal capsule. The internal capsule functions as a two-way tract pathway connecting the cortex to the subcortical structures involved in the motor and reward pathways. Subpanel e shows the fimbria of the fornix. The fornix is the main output of the hippocampus to subcortical structures such as the hypothalamus, thalamus, amygdala, and cingulate gyrus. The components of the white matter tracts are glial cells, the 'glue' that keeps the grey matter integrity intact. Majority of the tracts that are stained in this experiment are the myelinated axons of the regions mentioned above. The main producer of myelin in the brain is the oligodendrocyte. Proliferation of oligodendrocytes is rapid during the first three years of an individual's life, increasing from 7 billion to 28 billion [31]. There is also growing evidence that folate deficiency and genetic cases of CFD correlate with hypomyelination of white matter tracts [37]. Other components of the white matter that could also be stained are the fibrous astrocytes, radial glia (a form of astrocyte found in CNS white matter), and microglia. Astrocytes in the white matter provide structural and metabolic support with radial glia having a critical role during nervous system development. Microglia are support cells whose function is to remove damage and debris within the CNS.

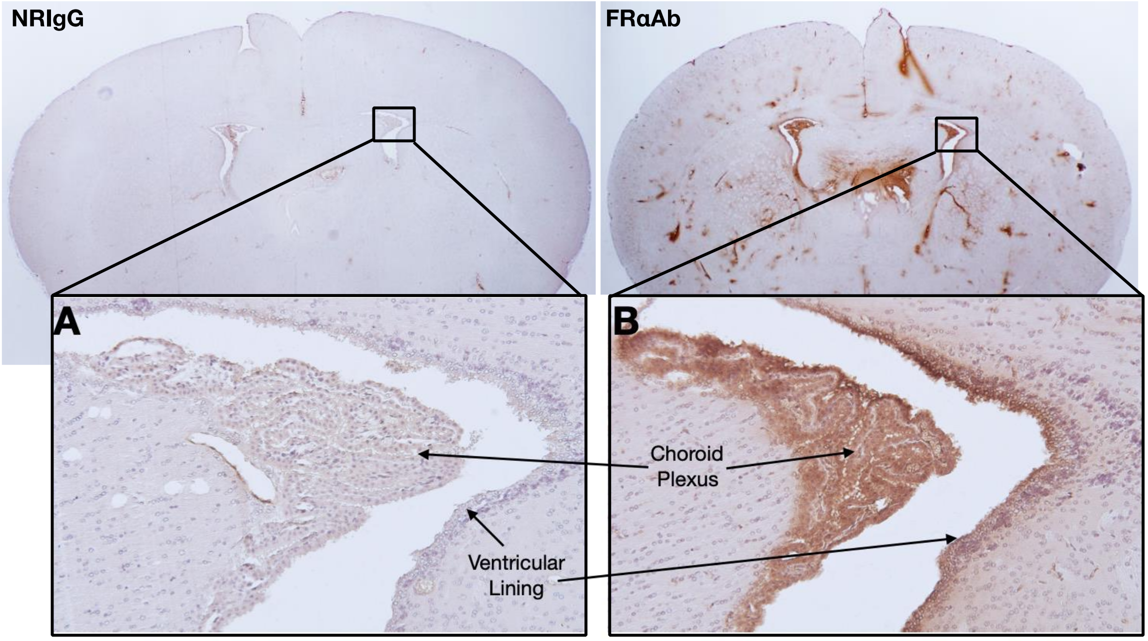

Supplementary Figure S2: Localization of NRIgG (A) and FRαAb (B) in PND23 brain vasculature, choroid plexus, and ventricular lining. Subjects used for this study were given 2 I.P. injections of antibody; the first injection 16 hours before the second injection, and the time of sacrifice 6 hours after the second injection.
